# Inhibition of the RIPK4 enhances suppression of human melanoma growth through vitamin D signaling

**DOI:** 10.1016/j.mce.2025.112603

**Published:** 2025-06-07

**Authors:** Bartlomiej Olajossy, Andrzej T. Slominski, Agnieszka Wolnicka–Glubisz

**Affiliations:** a Department of Biophysics and Cancer Biology, Faculty of Biochemistry, Biophysics and Biotechnology, Jagiellonian University, Gronostajowa Street 7, 30-387, Krakow, Poland; b Doctoral School of Exact and Natural Sciences, Jagiellonian University, Krakow, Poland; c Department of Dermatology, University of Alabama at Birmingham, Birmingham, AL, USA; d Veteran Administration Medical Center, Birmingham, AL, USA

**Keywords:** RIPK4, Melanoma, Spheroids, Calcitriol, VDR, CYP24A1

## Abstract

Downregulation of Receptor-Interacting Protein Kinase 4 (RIPK4) inhibits NF-κB and Wnt/β-catenin signaling in melanoma and xenograft growth in mice. The active form of vitamin D3 (1,25-D3), in addition to regulating calcium and phosphate metabolism in humans through the vitamin D receptor (VDR), can inhibit the NF-κB signaling pathway and can affect the proliferation and differentiation of normal and malignant cells, including melanoma. An hyperactive NF-κB pathway maintains the malignant behavior of melanoma, which can be influenced by both RIPK4 and activated VDR. As their interactions affecting the response to 1,25-D3 in melanoma have not been studied, we tested whether downregulation of RIPK4 affects the sensitivity of melanoma cells to 1,25-D3. Our results have shown that both siRIPK4 and CRISPR/Cas9-mediated RIPK4 knockout increase VDR expression in melanoma cells. Furthermore, a decrease in CYP24A1 expression and an increase in 1,25 D3-induced VDR levels were observed in cells with RIPK4 downregulation. Treatment with 1,25- D3 of RIPK4.KO cells, compared to their wild-type counterparts, significantly reduced proliferation in 2D and 3D culture (MTT or ATP assay) and decreased p-p65 and cyclin D1 levels in melanoma cells. These results indicate that RIPK4 knockout may enhance the therapeutic efficacy of 1,25-D3 against melanoma, which encourages further studies on targeting RIPK4 signaling for anti-melanoma effects in preclinical models.

## 1. Introduction

Receptor-Interacting Protein Kinase 4 (RIPK4) is a member of the RIP family and a known regulator of cell-cell adhesion in epidermal development and homeostasis (Fortugno et al., 2022; Kwa et al., 2014; Urwyler-Rösselet et al., 2023). Recent studies, have shown that disruption of RIPK4 expression, both downstream and upstream, has been linked to the tumorigenesis of in several experimental models (Gong et al., 2018; Heim et al., 2015; Liu et al., 2018, 2021; Shan et al., 2024). The RIPK4 functions in organs, such as skin, depend on cell type (epithelial or neural crest origin). In normal skin, high expression of this kinase is found in keratinocytes, while in squamous cell carcinoma (SCC) belonging to non-melanoma skin cancers (NMSC), its levels decrease (Xu et al., 2022). In contrast, in melanomas, which develops by malignant transformation of melanocytes (melanin pigment producing cells of neural crest origin), it demonstrates an oncogenic potential (Madej et al., 2021).

The global analysis of gene expression changes after RIPK4 silencing (*GSE 263112)* reveals a complex role for this kinase in regulating adhesion, migration, proliferation and inflammatory processes in melanoma cells, including an increase in expression of *VDR (fold change 2.18)* (Madej et al., 2024). RIPK4 involvement in melanoma progression is mediated through an activation of the NF-κB and Wnt/β-catenin, but without a connection with the BRAF/MEK/ERK signaling cascade (Madej et al., 2021, 2023, 2024; Wronski et al., 2024). However, treatment with BRAF inhibitors such as dabrafenib and vemurafenib reduces the expression of this kinase (Madej et al., 2023). Moreover *in vivo*, RIPK4 sustains xenograft growth in mice (Wronski et al., 2024). Recently, we have shown that downregulation of RIPK4 expression decreases ABCG2 expression and increases melanoma cell susceptibility to cisplatin and doxorubicin (Olajossy et al., 2024).

VDR, a nuclear steroid hormone receptor activated by 1,25-dihydroxy-vitamin D3 [calcitriol, 1,25-D3], affects proliferation and differentiation of normal and cancer cells, including melanoma (Slominski A.T. et al., 2024; Slominski et al., 2018), and may be a prognostic marker of melanoma (Brożyna et al., 2020; Brozyna et al., 2011; Slominski A.T. et al., 2024; Slominski R.M. et al., 2024). VDR may also act as a melanoma suppressor gene (Podgorska et al., 2021). The potential use of vitamin D and its analogues as an adjuvant treatment for melanoma has been indicated (Slominski R.M. et al., 2024). Interestingly, vitamin D3 hydroxyderivatives have been shown to inhibit the NF-κB signaling pathway in human keratinocytes and melanoma cells (Janjetovic et al., 2011, 2010; A. T. Slominski et al., 2024). In the skin, expression of both VDR and RIPK4 in epidermal keratinocytes remains high (Brozyna et al., 2011; Urwyler-Rösselet et al., 2023). RIPK4 and VDR have opposing functions in melanoma and both proteins differentially affect NF-κB signaling. The overactive NF-κB pathway maintains the malignant behavior of melanoma (Khan et al., 2024; Thu et al., 2011) that is associated with cell proliferation, and may serve as a molecular target for anticancer therapy and vitamin D action (Cornice et al., 2024; Janjetovic et al., 2011; Slominski A. T. et al., 2024).

Herein we studied if downregulation of RIPK4 can affect the expression of the VDR and the response of melanoma cell to 1,25-D3 treatment.

## 2. Results

### 2.1. RIPK4 downregulation increases VDR expression in melanoma cells

The RIPK4 expression in metastatic melanoma is high while being heterogeneous (Madej et al., 2021, 2023). In contrast, VDR expression *in vivo* decreases during the progression of melanocytic lesions and melanoma (Brozyna et al., 2011), but in different cell lines cultured *in vitro* levels of its expression remain heterogeneous (Slominski A.T. et al., 2012; Wronski et al., 2024).

**Fig. 1.**
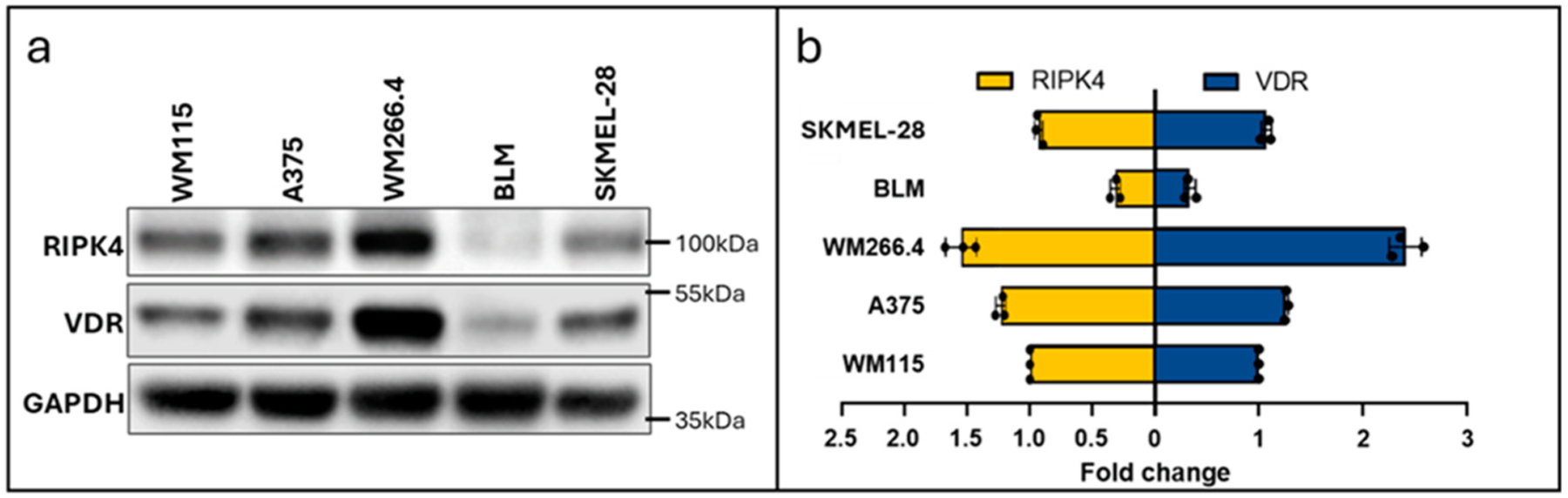
The level of RIPK4 in relation to VDR in various melanoma cell lines. Correlation of RIPK4 and VDR in human melanoma cells. (a) Protein level of RIPK4 and VDR in melanoma cells by Western blot. GAPDH was used as a loading control. (b) Fold change was counted relative to RIPK4 and VDR expression in melanoma lines, n = 3.

To evaluate the corelation of *RIPK4* with *VDR* in melanoma cells we have used human cell lines in which expression for both proietins were analysed using Western blot (WB) technique. Fig. 1a shows the expression o*f* RIPK4 with VDR in different melanoma lines, with the highest levels for both proteins in WM266.4 cells, and the lowest expression in BLM cells. The Pearson coefficient shows a strong correlation between the VDR and the RIPK4 protein expression in melanoma cell lines *(r* = *0.92, p* < *0.001*) (Fig. 1b).

**Fig. 2.**
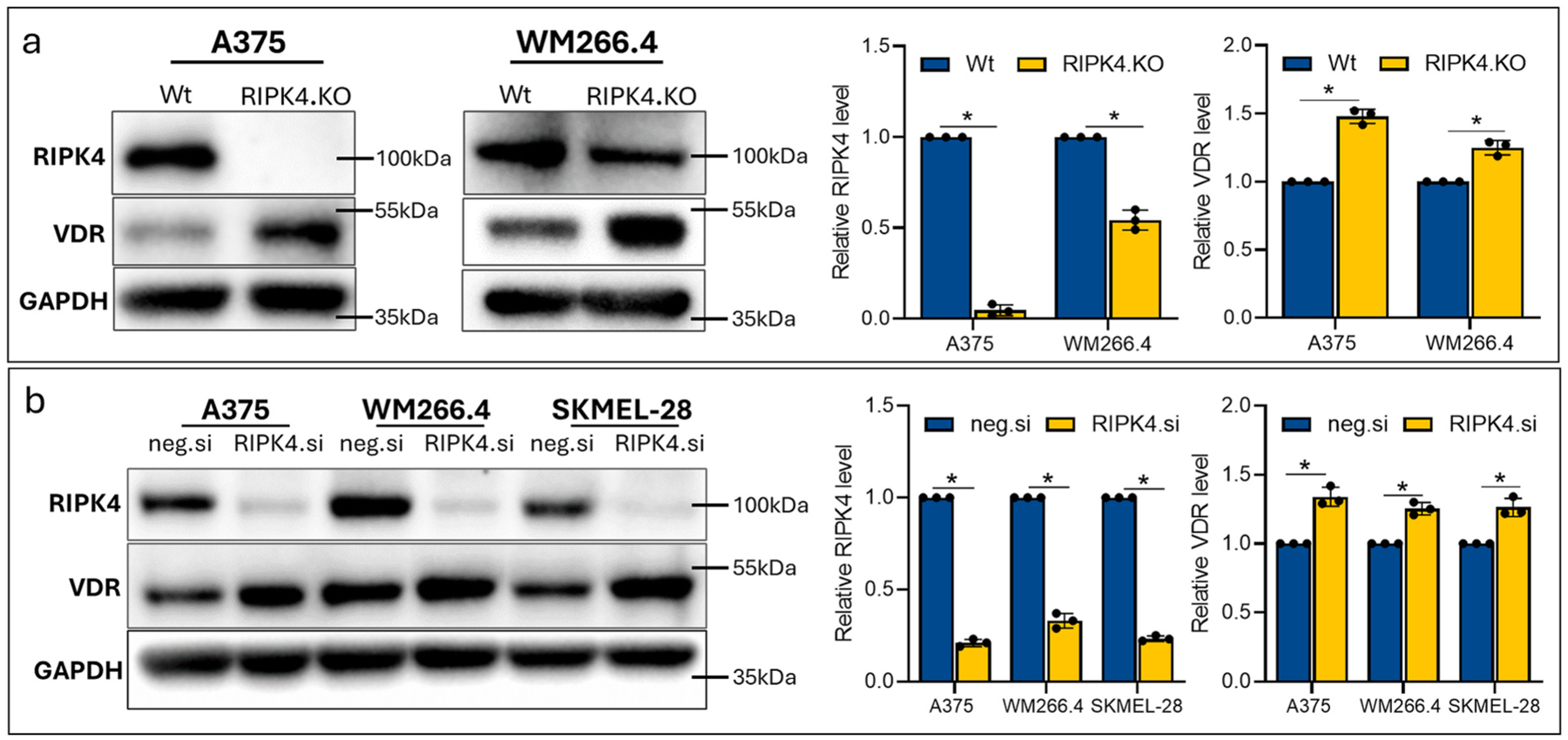
Effect of RIPK4 downregulation on VDR expression in melanoma cells. (a) Stable downregulation of RIPK4. The level of VDR in A375^RIPK4.KO^, WM266.4^RIPK4.KO^ cells and their parental lines (Wt). (b) RIPK4 silencing in cells transfected with RIPK4.si RNA, or neg.si RNA and analysed after 48 h by Western blot. GAPDH was used as a loading control. Densitometry (mean ± SD). n = 3. * indicates *p* <*0.05*.

To directly address the role of RIPK4 in VDR expression in melanoma cells, we have used previously established by us lines with stable RIPK4 knockout (A375^RIPK4.KO^ and WM266.4^RIPK4.KO^) (Wronski et al., 2024). Fig. 2a shows that the VDR expression in both A375^RIPK4.KO^ and WM266.4^RIPK4.KO^ cells is higher than in the corresponding wild-type cells.

In addition, we have downregulated RIPK4 expression using specific siRNA as described in (Madej et al., 2021; Olajossy et al., 2024). The silencing of RIPK4 in A375, WM266.4 and SKMEL-28 cells using RIPK4-specific small interfering RNAs (siRNAs) (by 70–80 % of the control level) also increases VDR expression by 20–30 % (Fig. 2b). Since silencing or downregulation of RIPK4 in cells increases VDR expression in A375 and WM266.4, we have tested whether these cells should are more sensitive to vitamin D treatment.

### 2.2. RIPK4 downregulation enhances sensitivity of melanoma cells towards 1,25-D3 treatment

**Fig. 3.**
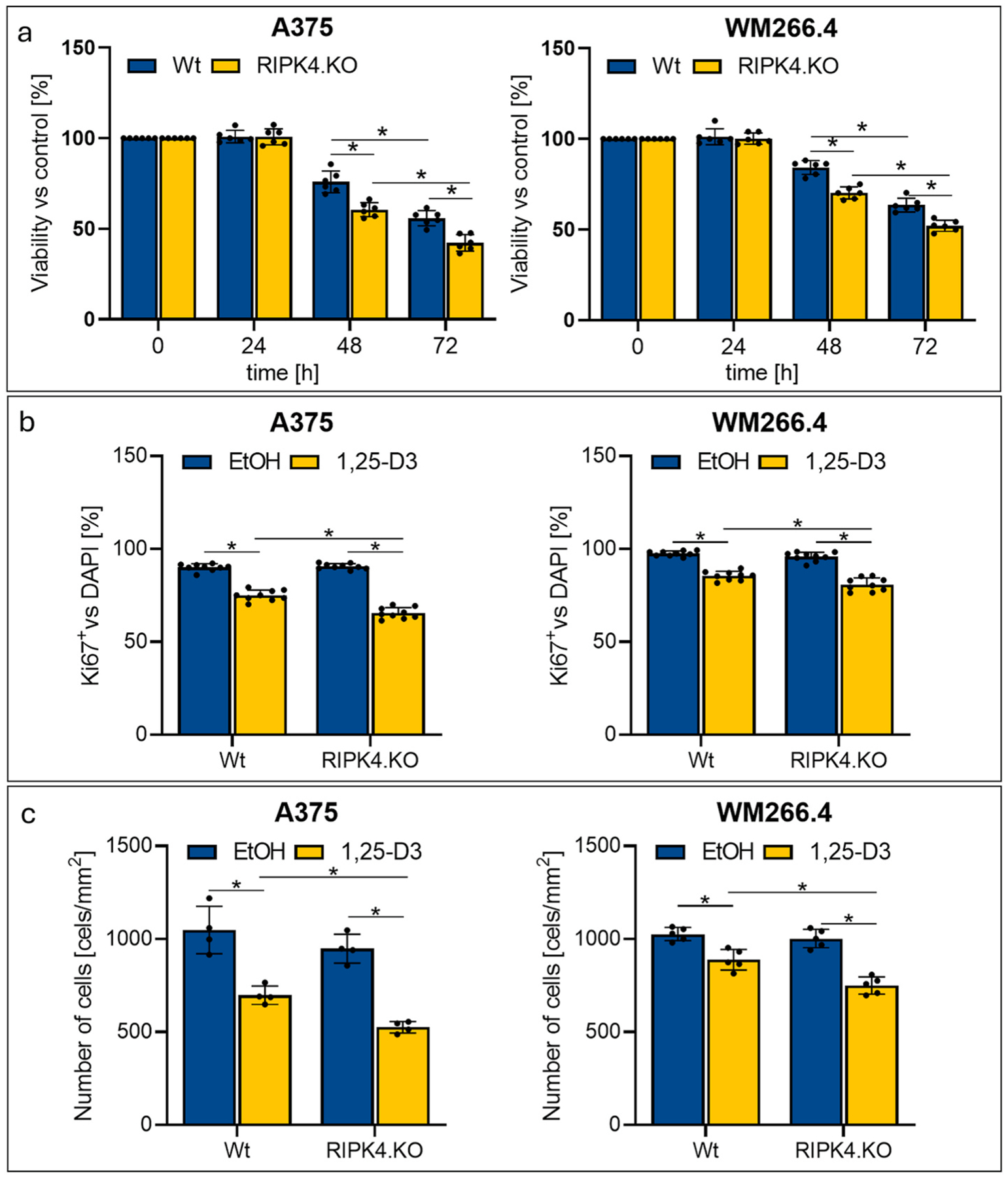
Downregulation of RIPK4 enhances suppression of human melanoma proliferation by 1,25-D3 (100 nM). Treated and untreated A375^RIPK4.KO^ and WM266.4^RIPK4.KO^ were compared with wild type controls (Wt). (a) Viability of the cells was assessed with MTT assay at indicated time, n = 6 (three biological replicates in duplicate). (b) Ki67 positive cells 48 h after the treatment. DAPI stained cells were defined as 100 %. (c) Number of cells 48 h after the treatment. Graph bars represent the mean ± SD of three biological replicates in triplicate, n = 9. * Indicates *p* < *0.05*.

We investigated the sensitivity of A375^RIPK4.KO^ and WM266.4^RIPK4.KO^ cells to the action of active form of vitamin D3 (1,25-D3). As excepted 1,25-D3 (100 nM) significantly (*p* < *0.05*) decreases number of viable melanoma cells 48–72 h after the treatment, and the effect is enhanced in A375^RIPK4.KO^ and WM266.4^RIPK4.KO^ cells in comparison to their wild type counterparts as measured by MTT test (Fig. 3a). This has been further validated by Ki67 staining, which shows that 1,25-D3 reduces significantly (*p* < *0.05*) the number of proliferating (Ki67+) cells by 33 % in A375^RIPK4.KO^ vs. 23 % in A375 ^wt^ 48 h after treatment, and by 19 % in WM266.4^RIPK4.KO^ vs. 14 % in WM266.4 ^wt^ (Fig. 3b–and Figure S1, S2 in supplementary materials). In addition, DAPI staining confirmed the lower number of cells after 1,25-D3 treatment compared to untreated cells (Figs. S1 and S2). Also, cell counting revealed that 1,25-D3 reduces significantly (*p* < *0.05*) their number by 45 % in A375^RIPK4.KO^ vs. 33 % in A375^wt^ 48 h after treatment, and by 25 % in WM266.4^RIPK4.KO^ vs. 13 % in WM266.4^wt^ (Fig. 3c). Thus, four independent tests demonstrate that RIPK4 downregulation enhances sensitivity of melanoma cells towards 1,25-D3 treatment. The observed more pronounced effects of RIPK4 knockout in A375 cells as compared to WM266.4, which may be secondary to more complete deletion of RIPK4 in A375 cells in comparison to WM266.4 cells (Fig. 2a).

**Fig. 4.**
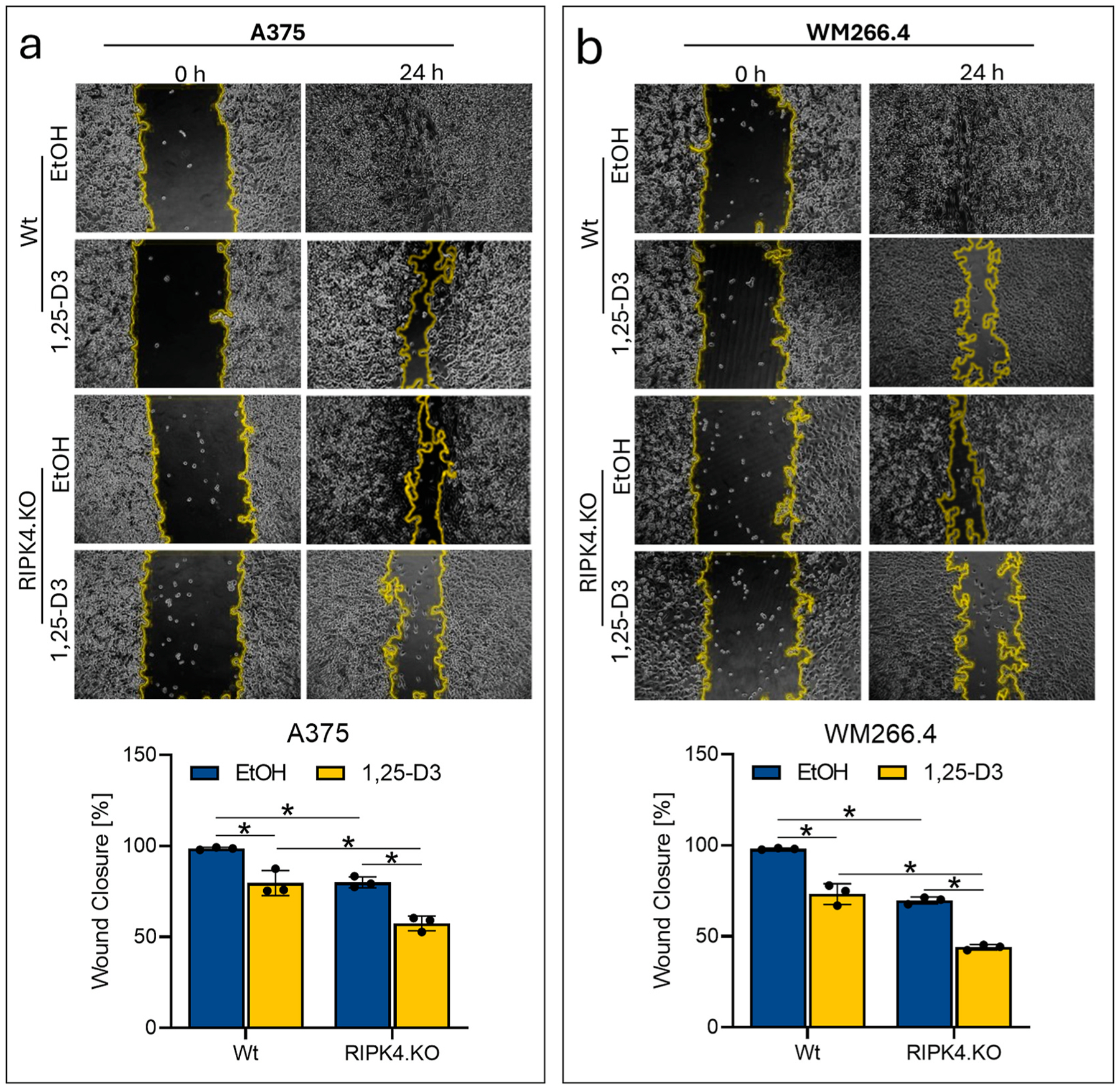
Downregulation of RIPK4 enhances suppression of human melanoma movement by 1,25-D3 (100 nM). Cells were pretreated with mitomycin C for 3 h to inhibit cells proliferation. (a, b) Treated and untreated A375^RIPK4.KO^ (a) and WM266.4^RIPK4.KO^ (b) were compared with wild type controls (Wt). Wound healing, n = 3. Pictures of the scratch area at 0 and 24 h (upper panel). Graph bars represent the mean ± SD of three biological replicates. * Indicates *p* < *0.05*.

Next, we testes the migration of 1,25-D3 treated and untreated RIPK4 KO type and Wt cells using the scratch assay. Movement of cells into the scratch area was evaluated over 24 h of incubation with 1,25-D3 at 10^−7^ M or the ethanol vehicle as control. Fig. 4 shows significant (*p* < *0.05*) inhibition of cell movement under the influence of 1,25-D3 regardless of the cell type. Again, the strongest effect of 1,25-D3 treatment is observed in RIPK4.KO cells (44 % for A375 and 58 % for WM266.4, vs. Wt, 18 %, 30 %, respectively).

### 2.3. 1,25-D3 induced upregulation of VDR and RORγ in enhanced in RIPK4.KO cells

**Fig. 5.**
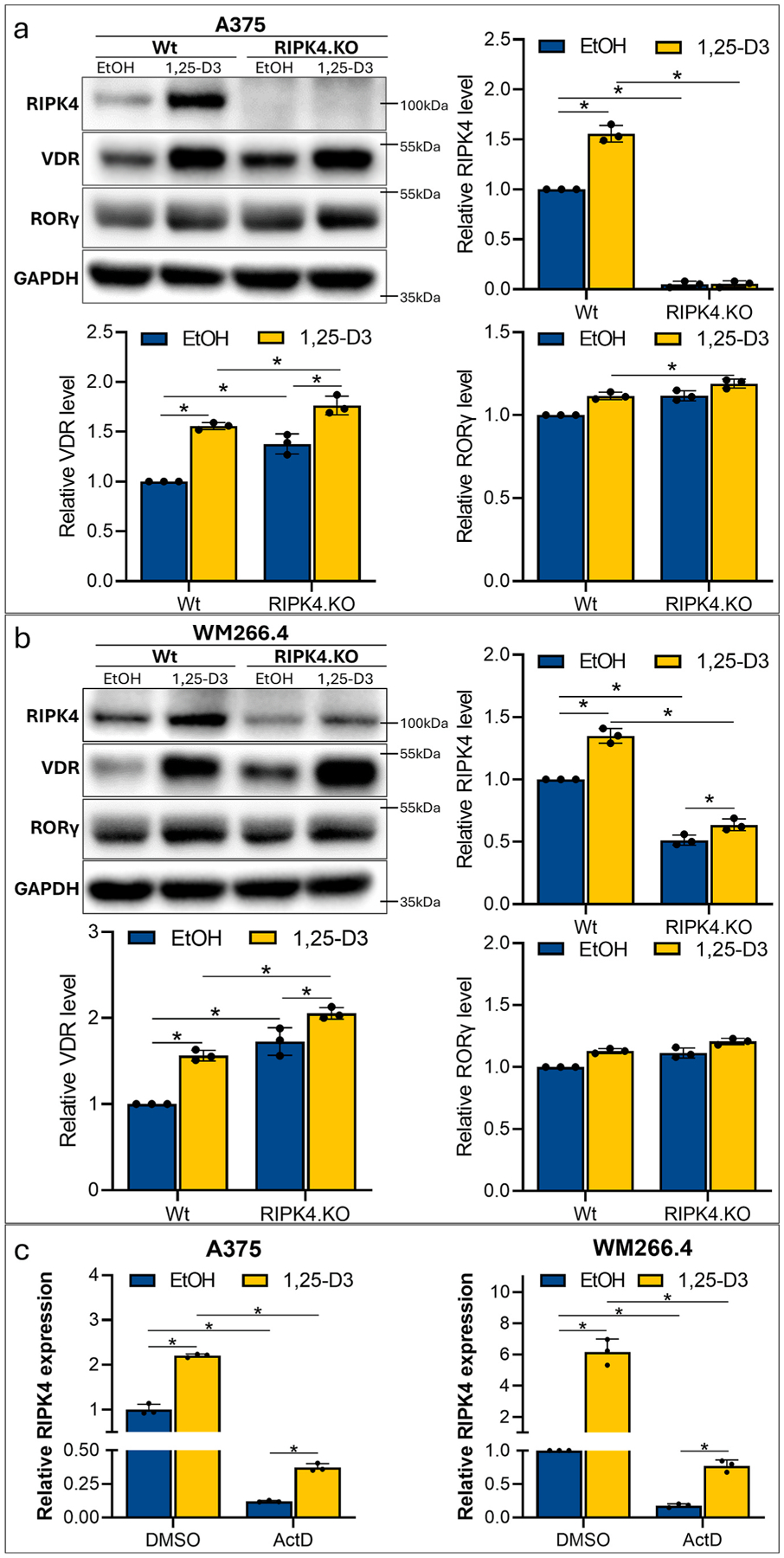
Effect of RIPK4 downregulation on VDR and RORγ protein levels induced by 1,25-D3 in A375^RIPK4.KO^ and WM266.4^RIPK4.KO^ and wildtype controls. (a) The level of selected protein by Western blot in melanoma cell lines 24 h after 1,25D*3* or EtOH as control. GAPDH was used as a loading control. (b) Protein levels of selected proteins by densitometry (mean ± SD). n = 3. (c)-A375 and WM266.4 cells were pretreated with 5 μg/ml actinomycin D (Act D) or DMSO for 1 h, followed by the treatment with 1,23-D3. Total RNA was isolated 6 h post treatment and Q-RT-PCR was performed. The RIPK4 transcript level was normalized to GAPDH transcript. Untreated cells served as a control. (mean ± SD). n = 3. * Indicates p < *0.05*.

Vitamin D hydroxyderivatives can exert anti-cancer activities acting as agonists on the VDR or can act as inverse agonists on retinoid related orphan receptors γ and α (RORγ and RORα) (Slominski A.T. et al., 2024; Slominski A. T. et al., 2014). Fig. 5a and b shows that 1,25-D3 significantly increases the expression of VDR and RIPK4 in melanoma cells 24 h after the treatment. These effects were more pronounced in RIPK4.KO cells than in Wt counterparts. The effect was not significant or minimal as relates to RORγ, while RORα remained below detectability level (data not shown). These results suggest significant role for the VDR in the observed effects. To examine transcript levels coding for *RIPK4* at the following time points: 3, 6, and 24 h after 1,25-D3 treatment, qRT-PCR was performed. Statistically significant up-regulation of *RIPK4* mRNA was observed in cells collected 6 and 24 h after the treatment similarly to *VDR* mRNA (Fig. S5 in supplementary materials). To investigate whether 1,25-D3 treatment affects the stability of *RIPK4* transcript, we pretreated A375 and WM266.4 melanoma cells with actinomycin D (Act D), an inhibitor of transcription, 1 h prior to the treatment. Fig. 5c shows that *RIPK4* mRNA levels decreases after preincubation with Act D, suggesting the transcript instability. The different level of *RIPK4* transcripts in Act D-treated cells with and without 1,25-D3 confirms *de novo* synthesis. In addition, bioinformatics analysis confirmed occurrence of the VDRE in the promoter region of the RIPK4 gene (*p* < *0.0001*; Table S1).

### 2.4. RIPK4 downregulation by Crisp/Cas9 decrease expression of CYP24A1 induced by 1,25-D3

**Fig. 6.**
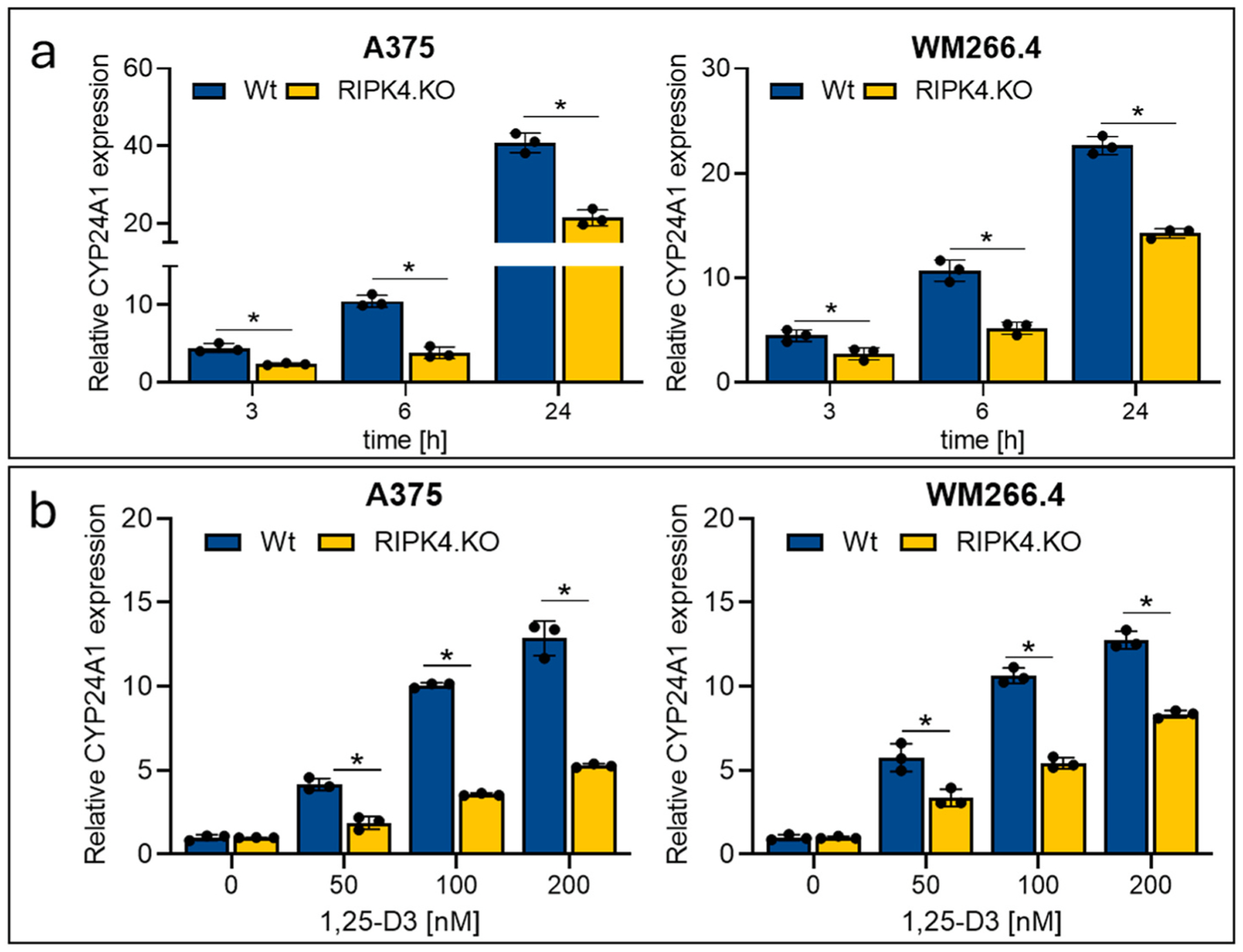
Transcript level of *CYP24A*1 in A375^RIPK4.KO^, WM266.4^RIPK4.KO^cells and their parental, wild type cells (Wt) untreated and treated with 1,25-D3 or EtOH (untreated control) normalized to *GAPDH*; n = 3. (a) Time dependent (3–24 h) after the treatment with 1,25-D3; 100 nM normalized to untreated control; (b) dose (50, 100, 200 nM) effect after 6 h of treatment. Each bar represents the mean ± SD. n = 3. * Indicates *p* < *0.05*.

Next, we investigated whether RIPK4 kinase influences 1,25-D3 metabolic inactivation. CYP24A1 is the main enzyme inactivating 1,25-D3 of which expression is positively controlled by the VDR (Holick, 2003). As expected, we observed increase of *CYP24A1* expression in time and dose dependent manner upon treatment with 1,25-D3 (Fig. 6a and b). Fig. 6 a, b shows that knocking out of RIPK4 in melanoma cells inhibits 1,25-D3 induced expression of *CYP24A1*.

### 2.5. RIPK4 dependent response to 1,25-D3 is regulated by NF-κB

**Fig. 7.**
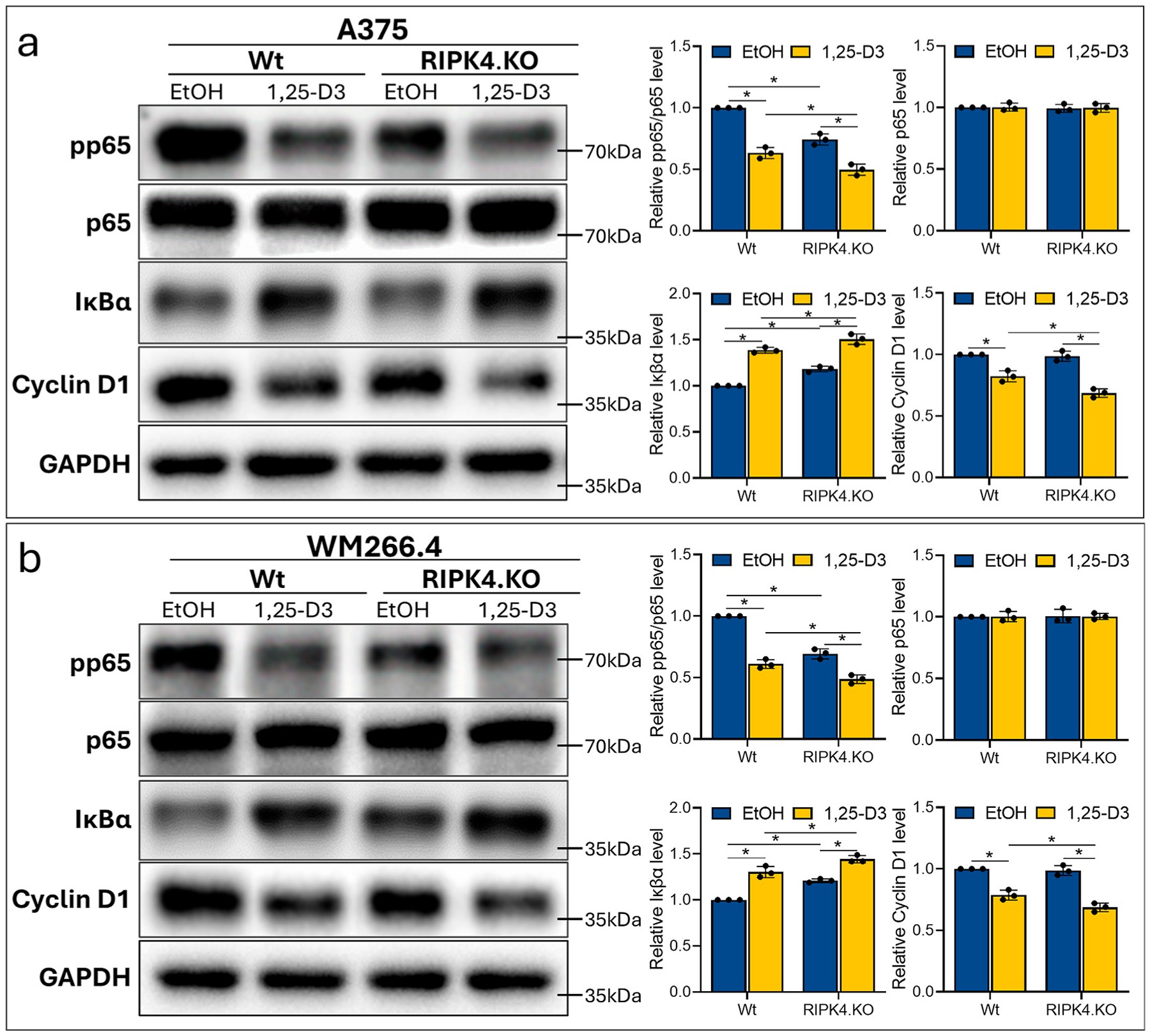
Effect of RIPK4 downregulation on the activity of NF-κB pathway inhibited by 1,25-D3 in A375^RIPK4.KO^ and WM266.4^RIPK4.KO^ and Wt controls. (a) The level of selected protein by Western blot in melanoma cell lines 24 h after 1,25-D3 or EtOH as control. GAPDH was used as a loading control. (b) Protein levels of selected proteins by densitometry (mean ± SD). n = 3. * Indicates *p* < *0.05*.

Biologically active forms of vitamin D downregulate the NF-κB pathway via inhibition of the nuclear translocation of the p65 NF-κB subunit to the nucleus with its accumulation in the cytoplasm and inhibition of NF-κB binding to DNA (Janjetovic et al., 2011). Szeto found that cells lacking the VDR exhibit increased NF-κB activity due to the reduction in IκBα levels and the lack of VDR-p65 interaction (Szeto et al., 2007). Chen show that VDR physically interacts with IκB kinase β (IKKβ) to block NF-κB activation (Chen et al., 2013). 1,25-D3 arrests p65 nuclear translocation, blocks NF-κB DNA binding, increases IκBα levels, or stabilizes IκBα. Here we have shown that while 1,25-D3 inhibit NF-κB activation and cyclin D1 in both A375 and WM266.4 wild-type control cells, the degree of the effect is affected by knocking out the RIPK4 (Fig. 7a and b). 1,25-D3 reduces the proteins level of p-p65 and cyclin D1 by 50 % and 31 % in A375^RIPK4.KO^ vs. 37 %, 18 % in A375 ^wt^ 24 h after treatment, and by 51 %, 32 % in WM266.4^RIPK4.KO^ vs. 39 %, 22 % in WM266.4^Wt^. This was accompanied by an in the level of Iκβα protein by 1,25-D3 by 50 %, in A375^RIPK4.KO^ vs. 39 %, in A375^wt^ and by 44 %, in WM266.4^RIPK4.KO^ vs. 30 %, in WM266.4^wt^

### 2.6. RIPK4 downregulation and 1,25-D3 inhibits spheroid formation

**Fig. 8.**
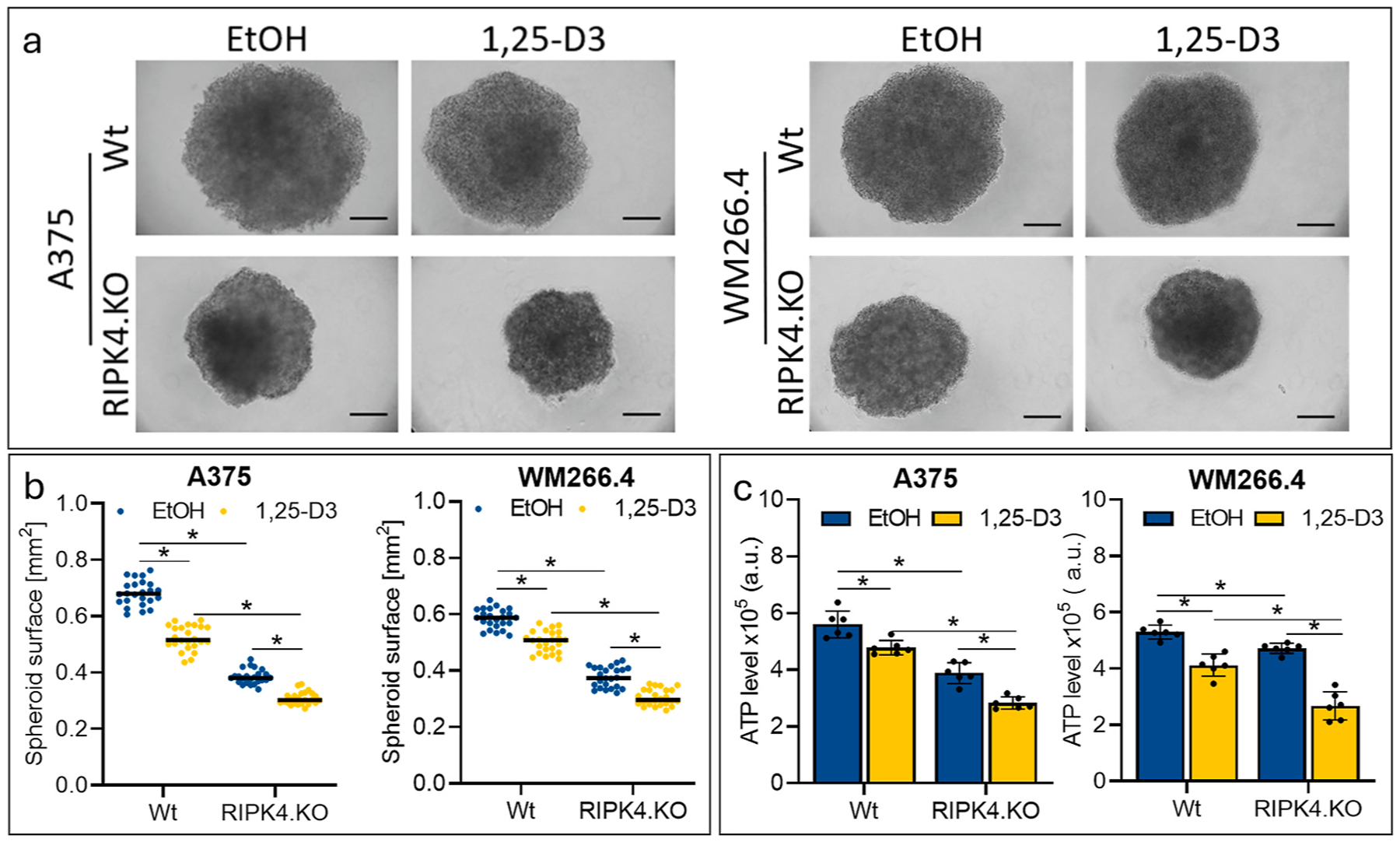
Effect of RIPK4.KO and 1,25-D3 on spheroid formation from A375 and WM266.4 cells. (a) Photographs of spheroids 5 days after seeding in a hanging drop in Opti-MEM in the presence of 1,25-D3 (100 nM) or EtOH (0.1 %) as control (0) cells. (b) Diameter and area analysis normalized to control. 8 spheroids each were collected per sample, three biological replicates (total n = 24). The scale represents 0.25 mm. (c) ATP by CellTiterGlo in 3D. n = 6. * Indicates p *< 0.05*.

To mimick *in vivo* conditions, we tested the effect of RIPK4 knockout and of 1,25-D3 using a 3D model. Spheroids were analysed after 5 days. As shown in Fig. 8a and b, RIPK4 knockout cells form smaller spheroids than wild-type control cells.

The formation of spheroids is also inhibited by the addition of 1,25-D3 to the medium in both Wt and RIPK4.KO cells of both lines (Fig. 8b). 1,25-D3 reduces spheroid formation by 24 %, in A375^RIPK4.KO^ vs. 11 %, in A375^wt^ 5 days after treatment and by 11 %, in WM266.4^RIPK4.KO^ vs. 4 %, in WM266.4^Wt^. However, RIPK4.KO also affects spheroid size. Spheroids formed from A375^RIPK4.KO^ cells are 44 % smaller than Wt, and WM266.4^RIPK4.KO^ cells are 34 % smaller, vs.Wt. Taken together, the simultaneous deletion of RIPK4 as well as the addition of 1,25-D3 reduces spheroid size by 56 % for A375 cells and 48 % for WM266.4. This was accompanied by a reduction in ATP levels by 49 % in A375^RIPK4.KO^ vs.15 % in A375 ^wt^ 5 days after treatment and by 50 % in WM266.4^RIPK4.KO^ vs. 22 % in WM266.4 ^wt^ (Fig. 8c), when compared to untreated wildtype cells confirming that elimination of RIPK4 together with 1,25-D3 treatment is most effective in inhibiting cell proliferation.

## 3. Discussion

Recent advances have shown that vitamin D3 signaling can affect melanoma behavior and represent a convenient target for adjuvant therapy (R. M. Slominski et al., 2024). In search of molecules which can increase sensitivity of melanoma cells to 1,25-D3 treatment we focus on RIPK4 as an increase in VDR expression (fold change of 2.18) has been shown in a global analysis of gene expression changes after RIPK4 silencing (GSE 263112) (Madej et al., 2024). Moreover, NGS analysis of WM266.4 melanoma cells with RIPK4 silencing reveals a complex role for this kinase in regulating adhesion, migration, proliferation and inflammatory processes (Madej et al., 2024). Those process are also regulated by activated VDR (Muralidhar et al., 2019; Podgorska et al., 2021).

In this study, we show that 1,25-D3 increases levels of both VDR and RIPK4 in Wt melanoma cells.

To investigate the impact of RIPK4 on VDR expression, we performed gene silencing in two ways: by siRNA and through stable knockout using CRISPR/Cas9. Both silencing and knockout of RIPK4 in melanoma cells results in increase protein level of VDR. The treatment with 1,25-D3 decreases cell viability (MTT) and proliferation (Ki67, cell number, cyclin D1 level), migration (scratch assay) in 2D and 3D culture. In both models the effect was more pronounced in RIPK4.KO cells. Interestingly, our study showed that the effects of RIPK4 knockout are more pronounced in A375 as compared to WM266.4 cells. This is probably be secondary to complete deletion of RIPK4 in A375 vs. not fully complete in WM266.4 cells (Fig. 2a). The latter is secondary to a greater heterogeneity of the WM266.4^RIPK4.KO^ cell population (Wronski et al., 2024).

Similarly, Sertznig et al. (2009) showed that the expression of VDR was stronger in the 1,25-D3-sensitive melanoma cells such as MeWo and SKMEL-28, compared to 1,25-D3-resistant melanoma cell lines such as SKMEL-5 and SKMEL-25. They also showed that treatment with 1,25-D3 increased VDR expression in MeWo and SKMEL-28 cells but not in SKMEL-5 and SKMEL-25 cell lines. Quarni et al. have shown that RIPK1 decreases 1,25-D3-induced growth suppression (Quarni et al., 2017). We have recently shown that downregulation of RIPK4 expression decreases ABCG2 expression and increases the susceptibility of melanoma cells to cisplatin and doxorubicin (Olajossy et al., 2024). Silencing of RIPK4 through siRNA or knockout via the CRISPR-Cas9 system does not induce apoptosis in melanoma cells *in vitro* but reduces xenograft growth *in vivo* mouse model (Olajossy et al., 2024; Wronski et al., 2024). Therefore, we suggest that inhibition of RIPK4 can decrease the malignant potential of melanoma cells and leads to increased anti-melanoma activity of 1, 25-D3. These effects appear to be associated with an increased expression of the VDR, a main target for 1,25-D3 activity, and of which decreased expression can lead to more aggressive behavior of melanoma (Brożyna et al., 2020; Brozyna et al., 2011; Podgorska et al., 2021).

Our studies show that the knocking out of RIPK4 also affects the degradation of 1,25-D3. The key role of CYP24A1 is to shorten the side chain of the 1,25-D3 leading to its inactivation and prevention of the 1,25-D3 systemic calcemic effects. In addition, it has been proposed that overexpression of CYP24A1 results in an increased resistance to the antiproliferative action of 1,25-D3 (Fleet, 2008), and this enzyme has been considered as a putative proto-oncogene (Kósa et al., 2013; Sakaki et al., 2014). We found a decrease in *CYP24A1* expression in RIPK4.KO cells treated with 1,25-D3 compared to their Wt counterparts, which was time- and dose-dependent. This suggests that an inhibition of RIPK4 can increase 1,25-D3 bioavailability leading to an enhanced anti-melanoma activity. However, the mechanism of this unexpected phenomenon needs to be further investigated. The formation of a calcitriol/VDR/RORX complex is required for CYP24A1 transcriptional regulation. Such a complex requires binding to two functional VDREs within the CYP24A1 promoter, of which there are five in the human promoter (Milan and Ramkumar, 2024). The casein kinase 2 (CK2) also plays a role in regulating CYP24A1 expression, which has been shown in prostate cancer cells (Luo et al., 2013). CK2 is frequently dysregulated and overexpressed in various cancers, including melanoma. It plays a key role in cellular processes such as proliferation, cell cycle control and resistance to cell death, making it a potential target for anticancer therapy (Borgo et al., 2021). Elevated CK2 activity has been observed in metastatic melanoma, but there are no data on the relationship between RIPK4 and CK2 so far.

Mechanistically, it appears that interactions between RIPK4 and the active VDR may influence the 1,25-D3 responses through the NF-κB pathway. An overactive NF-κB pathway maintains the malignant behavior of melanoma (Olajossy et al., 2024; A. T. Slominski et al., 2024). Downregulation of RIPK4 impairs signaling pathways important for melanoma survival and progression, such as NF-κB (Madej et al., 2021, 2024) and Wnt/β-catenin (Wronski et al., 2024). These pathways are regulated by the VDR (Janjetovic et al., 2011, 2010; A. T. Slominski et al., 2024). Indeed, downregulation of RIPK4 leads to increased VDR expression and inhibition of NF-κB signaling that includes an increase of IκBα with lower levels of p-p65. This effect is more pronounced upon treatment with 1,25-D3.

Unexpectedly, 1,25-D3 increases RIPK4 at transcript and protein level in Wt cells, even though we observe an increase in VDR and inhibition of the NF-κB pathway. However, it should be noted, that the final biological effect achieved by 1,25-D3 is stronger when RIPK4 is absent. Moreover, our data revealed that increase of RIPK4 in melanoma cells treated with 1,25-D3 is due to *de novo* synthesis of transcripts. The phenomenon of stimulation of *VDR* gene expression in skin cells including melanomas has been reviewed (A. T. Slominski et al., 2024; R. M. Slominski et al., 2024). It is possible that RIPK4 acts similarly to RIPK1, which inhibits VDR transcriptional activity (Quarni et al., 2017). Inhibition of VDR transcriptional activity occurs independently of RIPK1 kinase activity and is likely to be mediated by a RIPK1-VDR pro-protein interaction that increases cytoplasmic retention of the VDR (Quarni et al., 2017). The close evolutionary relationship of gene expression between RIPK1 and RIPK4 and the homology of 10 of the 12 critical amino acid residues in both kinases (Lv et al., 2022) suggests that RIPK4, analogous to RIPK1, may affect cytoplasmic VDR retention. These possibilities await future experimental testing.

In conclusion, our study envisages RIPK4 depletion as a potential intervention strategy in melanoma and to enhance the potency of 1,25-D3 and its analogues in melanoma therapy. These possibilities await future experimental testing.

In summary, our studies project RIPK4 depletion as a potential strategy in melanoma intervention and to increase the potency of 1,25-D3 and its analogues in melanoma therapy.

## 4. Materials and methods

### 4.1. Reagents

Vitamin D3 (1,25-D3 Cat. No. D-1530), protease inhibitor cocktail (Cat. No. P8340), Polybrene (Cat. No. TR1003), RIPA buffer (Cat. No. R0278), Bicinchoninic Acid Protein Assay Kit (Cat. No. B9643), penicillin 150 U/mL, and streptomycin 100 μg/ml (Cat. No. P4333), Fluoroshield^™^ with DAPI (Cat. No. F6057), Actinomycin D (Cat. No. A9415) and Mitomycyn C (Cat. No. M0440) were from Sigma-Aldrich (St. Louis, MO, USA). The 12 % Bis-Tris gels—TGX^™^ FastCast^™^ Acrylamide Kit (Cat. No. 1610175) and Clarity Western ECL substrate (Cat. No. 1705060) were from Bio-Rad, USA. PVDF membranes (0.2 mm pore size; Cat. No. ISEQ00010) were from Millipore. Bovine serum (BSA; Cat. No. ALB001) was from BioShop, Burlington, ON, Canada, Fetal bovine serum (FBS; Cat. No. 10500064) and Opti-MEM (Cat. No. 31985–070) was from Gibco. CellTiter-Glo 3D Cell Viability Assay was from Promega (Wisconsin, USA). Mouse anti-Ki67 antibody conjugated to Alexa 647 (Cat. No. NBP2–2211AF647) was from Biotechne, USA). Ethanol, methanol, formaldehyde, and DMSO were purchased from POCH (Poland). Rabbit antibodies anti-RIPK4, p-p65, p65, IκBα, cyclin D1, GAPDH and goat HRP-conjugated anti-rabbit were from Cell Signaling Technology (Danvers, USA). Mouse anti-VDR antibody was from Santa Cruz (Dallas, Texas USA). Goat HRP-conjugated anti-mouse antibody was from BP Pharmingen, NJ, USA. All TaqMan primers and RORγ were purchased from Thermo Fisher Scientific/Invitrogen, RPMI-1640 culture medium with L-glutamine (Sartorius, Kostrzyn, Poland), total RNA Mini isolation kit (A&A Biotechnology, Gdansk, Poland), all primary antibodies and HRP conjugated anti-rabbit were from Cell Signaling Technology HRP conjugated anti-mouse was from BD Pharmingen.

### 4.2. Cell cultures and 1,25-D3 treatment

The experiments were performed on unpigmented human melanoma cells: BLM, SKMEL-28, A375, WM266.4 as described (Olajossy et al., 2024; Wronski et al., 2024). A375^RIPK4.KO^ and WM266.4^RIPK4.KO^ lines derived with a stable knockout of the RIPK4 gene obtained using the CRISPR/Cas9 system as described (Wronski et al., 2024). All cultures were conducted in RPMI-1640 culture medium with L-glutamine (Sartorius, Kostrzyn, Poland) supplemented with 10 % FBS (fetal bovine serum) and 1 % antibiotics (penicillin, streptomycin). The culture was cultivated at 37 °C, 80 % humidity and in an atmosphere enriched with 5 % CO_2_.

Cells were seeded 24 h before the treatment. Then we follow the Kosa a protocol and used the Opti-MEM medium instead of the culture one avoids indefinite concentrations of 1,25-D3 presence in serum (Kósa et al., 2013). 1,25-D3 was dissolved in absolute ethanol a stock solution (10^−4^ M), and further diluted with Opti-MEM medium (10^−7^ M). The culture medium was then replaced with medium containing 1,25-D3 and cells were incubated for 24–72 h. Actinomycin D (Sigma-Aldrich), a transcription inhibitor, was applied 1 h before 1,25-D3 treatment in the concentrations of 5 μg/ml (Bugara et al., 2017).

### 4.3. Viability assay in 2D and 3D culture

Cell viability in 2D culture was analysed suing the MTT assay as preciously describe (Madej et al., 2023; Olajossy et al., 2024) while in 3D culture by ATP assay. The spheroids along with the droplet-forming medium were supplemented with 30 μl of Opti-MEM and incubated at room temperature for 30 min in 96-well white-wall plates. Subsequently, 50 μl of CellTiter-Glo^®^ 3D Cell Viability Assay (Promega, Wisconsin, USA) was added and shaken for 5 min. After 25 min of incubation at room temperature, luminescence was measured using a BMG CLARIOstar reader (BMG Labtech, Germany).

### 4.4. Spheroid formation - 3D cultures

12 × 10^4^ cells were suspended in 600 μl Opti-MEM supplemented with 75 μl methylcellulose, 1,25-D3 (100 nM) or EtOH (0,3 %) as controls 5 ml of PBS was added to the bottom of 60 mm diameter dishes and then cell droplets (6 × 10^3^ cells/droplet) in a volume of 30 μl were placed on the lid. Twenty spheroids were created. The lids were inverted and placed over PBS to prevent the spheroids from drying out. The dishes were carefully placed in an incubator at 37 °C for 5 days. After this time, images were taken of the spheroids using an OPTIKA IM-3 microscope (OLYMPUS, IX73, Japan) for further analysis using a macro for ImageJ software designed for spheroids.

### 4.5. qRT-PCR

RNA was isolated from cells using the Total RNA Mini isolation kit (A&A Biotechnology, Gdansk, Poland) according to the protocol. The quality and concentration of the resulting RNA was checked on a BMG CLARIOstar reader (BMG Labtech, Germany) at wavelengths of 260 nm and 280 nm. 25-Hydroxyvitamin D-24-hydroxylase (*CYP24A1,* # Hs00167999_m1), *RIPK4* (Hs01062501_m1), Vitamin D Receptor (*VDR, #*Hs00172113_m1) gene expression levels were measured by RT-qPCR using a qTOWER3 thermocycler (Analitik Jena, Jena, Germany). Expression levels were normalized to the expression of *GAPDH (#*310884E). Relative expression was quantified using the 2^−ΔΔCt^ method.

### 4.6. Western blot

Cells were lysed in RIPA lysis buffer supplemented with a complete protease inhibitor cocktail (P8340, Merck-Sigma MI, USA). The protein concentration was determined using a bicinchoninic acid protein assay kit (#71287-M, Sigma) according to the manufacturer’s protocol. Western blots were performed as described (Olajossy et al., 2024). The following primary antibodies: specific for human RIPK4 (#12636), VDR (sc-13133), RORγ (PA5–23148), p-p65 (#3033), p65 (#4764), IκBα (#4812), cyclin D1 (#55506) followed by secondary antibodies: horseradish peroxidase (HRP)-conjugated anti-rabbit (#7074) or anti-mouse (#554002). GAPDH (#5174) was used as a loading control. Immunopositive bands were visualized using Clarity Western ECL substrate (Bio-Rad, California, USA) with a ChemiDoc detector (BioRad, Hercules, CA, USA). The intensity of the bands was quantified using ImageLab software 5.2.1 Original uncut and unprocessed scans of all blots are included in the raw data file.

### 4.7. Immunofluorescence staining for Ki67 and DAPI

The cells were washed with PBS solution with Ca^2+^ and Mg^2+^ ions and fixed with 3.7 % formaldehyde for 10 min at room temperature. To permeabilize the cell membrane, cells were incubated with 90 % methanol for 5 min. The cells were then washed with PBS buffer and incubated with 3 % BSA in PBS for 30 min. Subsequently, the cells were incubated for 1 h with mouse anti-Ki67 antibody conjugated to Alexa 647 (Biotechne, USA) at 1:40 dilution in blocking solution (3 % BSA in PBS). Additionally, cell nuclei were stained using Fluorshield^™^ with DAPI. Images were captured using a ImagEMX2 EM-CCD camera attached to a Axio Observer microscope (ZEISS, Oberkochen, Germany) and analysed using ImageJ.

### 4.8. Scratch assay

Cells were cultured in a 24-well plate until they reached 95 % confluence. When confluent, cells were pretreated for 3 h with mitomycin C (10 μg/ml) to inhibit cell proliferation. After 3 h, a scratch was created through the center of each well using the tip of an automated pipette. After the formation of scratches, the cells were washed with PBS, and the cells were incubated with 1,25-D3 at a concentration of 10^−7^ M or ethanol as a control. Microscopic images of each scratch were captured, and the rate of scratch closure was monitored at 6, 12, and 24 h, continuing until the scratches in untreated wild-type cells were fully closed. Migration analysis was performed using ImageJ software.

### 4.9. Bioinformatics analysis of the RIPK4 promoter region

Probability of occurrence of a VDRE (vitamin D response element) in the promoter region of the RIPK4 gene were tested using Find Individual Motif Occurrences (FIMO) tool (Grant et al., 2011) available at http://meme.sdsc.edu. The 2000 bp sequence upstream of RIPK4 promoter region downloaded from http://meme.sdsc.edu was used as the promoter regions to identify the VDRE-elements. The sequences for VDRE were downloaded from https://jaspar.elixir.no.

### 4.10. Statistical analysis

Statistical analyses were performed using GraphPad Prism software (version 9.0.0, GraphPad Software, La Jolla, CA, USA). The differences were assessed by ANOVA or two-tailed unpaired Student *t-test* with *p* < *0.05* considered as significant and labelled by an asterisk in the figures.

## 5. Limitations

Although we have shown that RIPK4 knockout enhances the therapeutic effect of 1,25-D3 in 2D and 3D *in vitro*, further studies in preclinical models are needed to confirm this effect in *vivo* system. This should also include clinicopathological studies in human biopsy/excision samples with correlation of its expression to clinical or pathological parameters of melanoma behavior or disease outcome. Such combined studies using animal models of melanoma and clinicopathological parameters can provide directions how to target RIPK4 in future research aimed to find optimal methods for melanoma treatment.

## 6. Conclusions

**Fig. 9.**
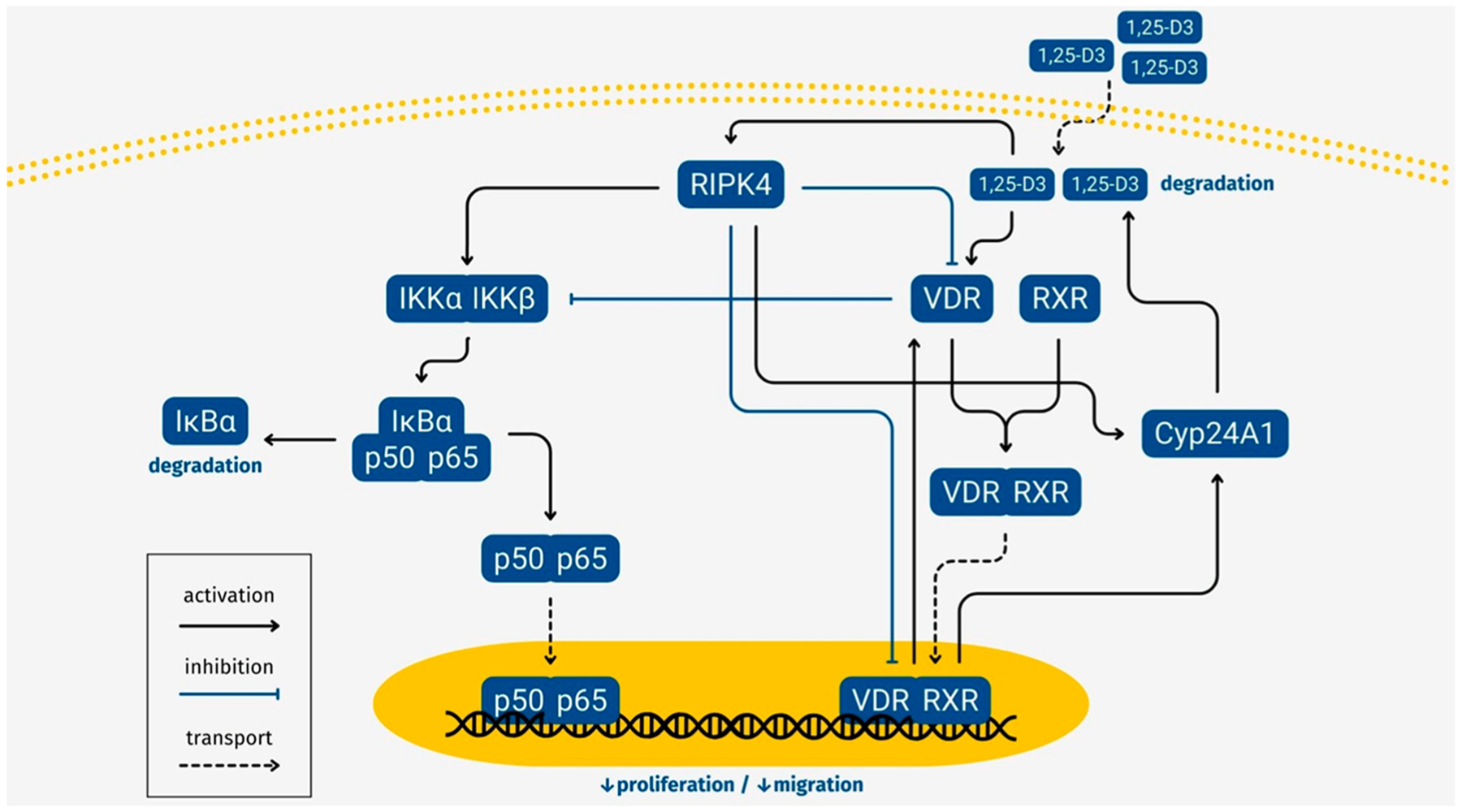
Proposed mechanisms of action of 1,25-D3 and RIPK4 on the vitamin D signaling in melanoma cells.

In conclusion, this is the first demonstration that RIPK4 plays a role in the regulation of VDR levels in melanoma cells, amplification of 1,25-D3 anti-melanoma effects and inhibition of CYP24A1, an enzyme inactivating 1,25-D3 (Fig. 9). Specifically, our results show that both transient and stable deletion of RIPK4 increases VDR expression in melanoma cells, demonstrating that RIPK4 plays a role in regulating VDR levels. Given our previous studies showing that RIPK4 participates in the regulation of melanoma adhesion, migration, proliferation and inflammatory processes, the present data suggest that RIPK4 inhibition may partially suppress the malignant behavior of melanoma by stimulating vitamin D signaling. These findings open new directions for research and development of new strategies in treatment of malignant melanoma.

## Supplementary Material

Supplementary material

Appendix A. Supplementary data

Supplementary data to this article can be found online at https://doi.org/10.1016/j.mce.2025.112603.

## Data Availability

Data are available at https://uj.rodbuk.pl.

## Data availability

Data will be made available on request.
