# Supplementary material for: Inhibition of the RIPK4 enhances suppression of human melanoma growth through vitamin D signaling
Source: Mol Cell Endocrinol. Author manuscript; Available in PMC 2026 Jun 7. (PMC12239225; doi:10.1016/j.mce.2025.112603)
Supplement: Supplementary material [file NIHMS2094702-supplement-Supplementary_material.docx]

Supplementary Materials

Inhibition of the RIPK4 enhances suppression of human melanoma growth through vitamin D signaling

Bartłomiej Olajossy ^a, b^, Andrzej Slominski ^c, d^ and Agnieszka Wolnicka-Glubisz ^a^ *

^a^ Department of Biophysics and Cancer Biology, Faculty of Biochemistry, Biophysics and Biotechnology, Jagiellonian University, Gronostajowa Street 7, 30-387 Krakow, Poland

^b^ Doctoral School of Exact and Natural Sciences, Jagiellonian University, Krakow, Poland

^c^ Department of Dermatology, University of Alabama at Birmingham, Birmingham, A, USA

^d^ Veteran Administration Medical Center, Birmingham, AL, USA

***** Correspondence: a.wolnicka-glubisz@uj.edu.pl; Tel.: +48-12-6646526

**
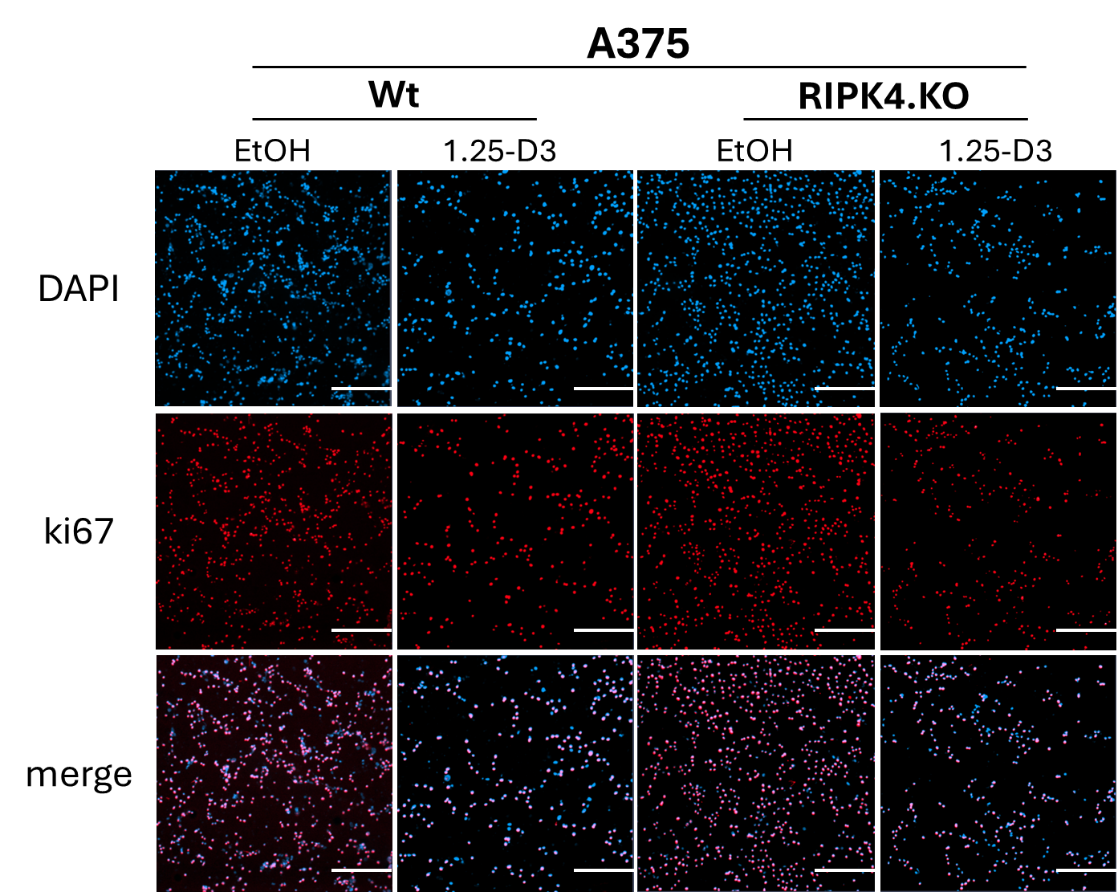
**

**Figure S1.** Immunofluorescence images of A375^Wt^ and A375^RIPK4.KO^ stained for Ki67 protein (red) and cell nuclei (blue, DAPI) 48 h after the treatment with 1,25-D3 (100 nM) or EtOH as a vehicle control. Scale bar = 200µm

*
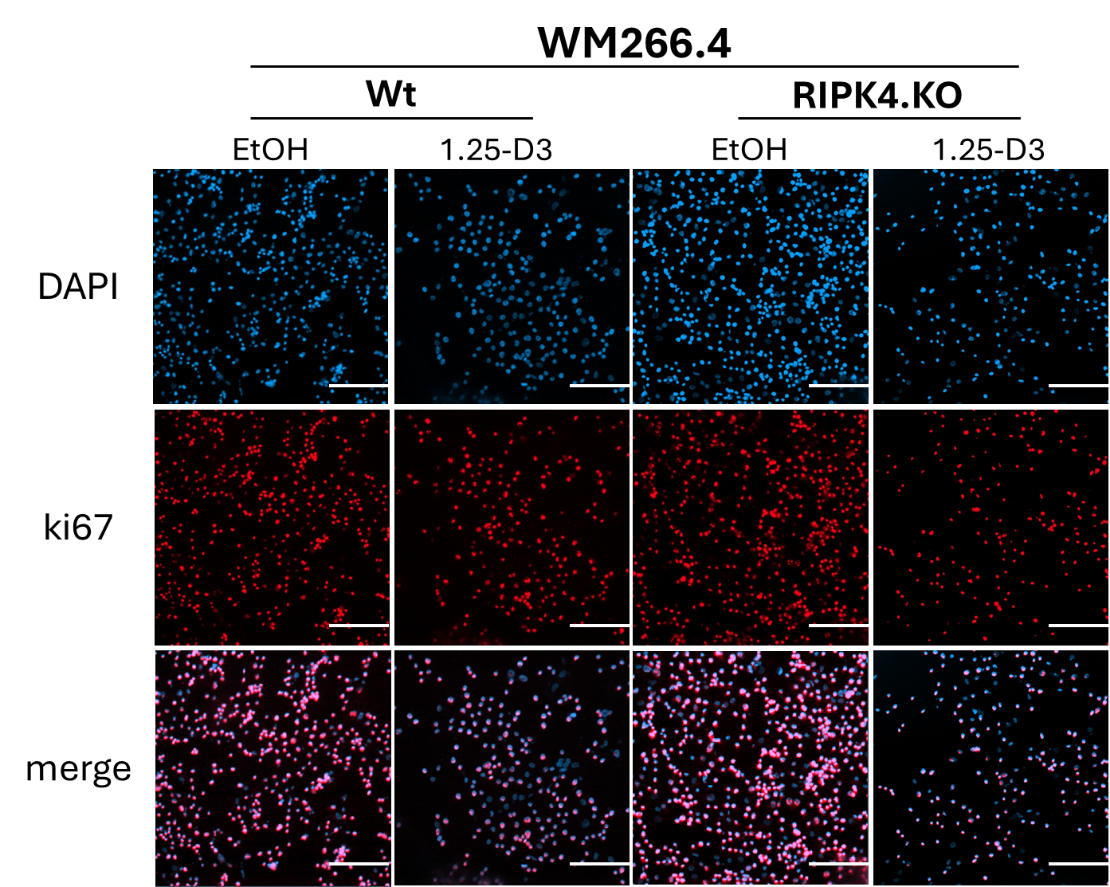
*

**Figure S2.** Immunofluorescence images of WM266.45^Wt^ and WM266.4^RIPK4.KO^ stained for Ki67 protein (red) and cell nuclei (blue, DAPI) 48 h after the treatment with 1,25-D3 (100 nM) or EtOH as a vehicle control. Scale bar = 200µm.

*
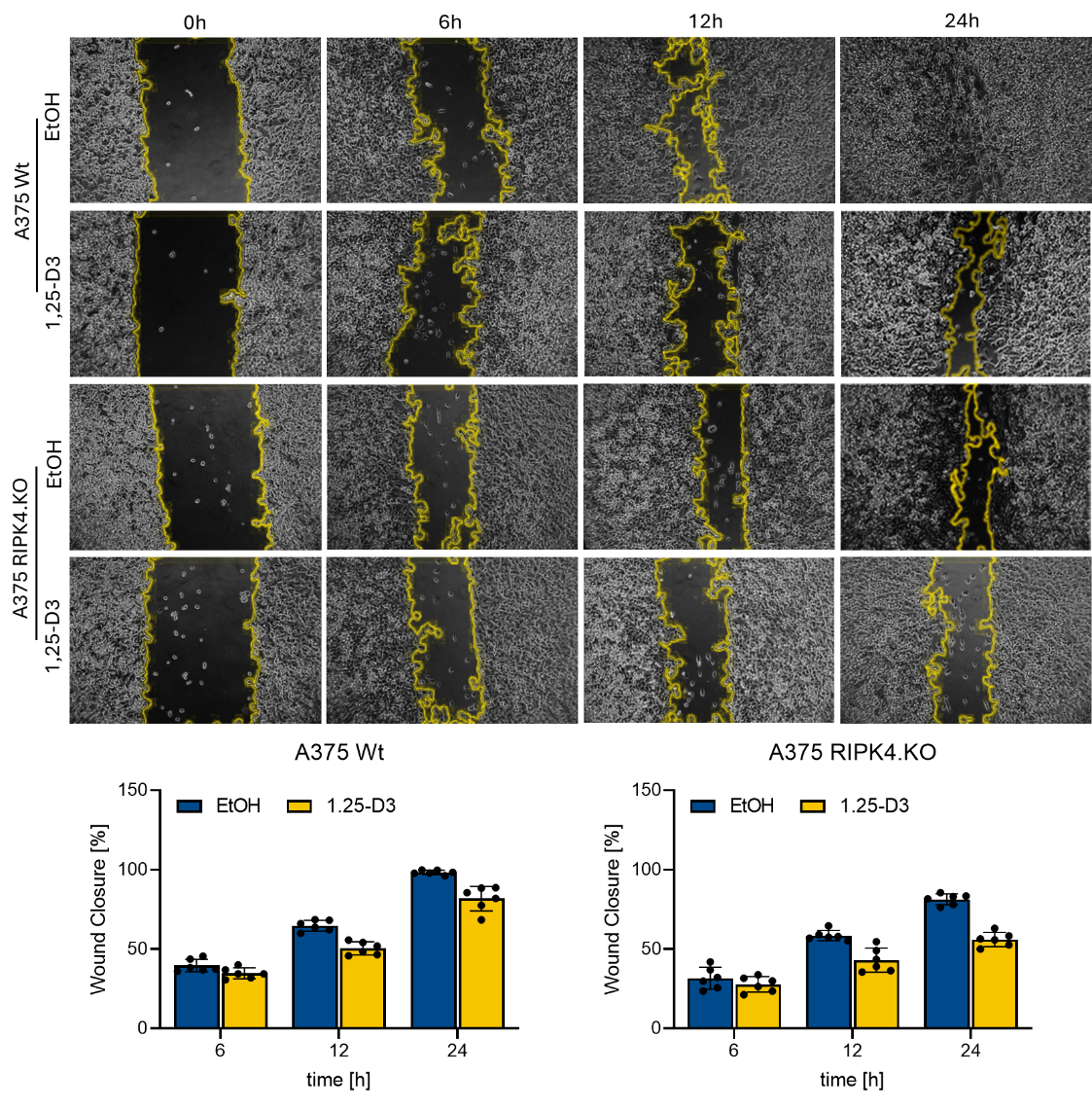
*

**Figure S3.** Images from *in vitro* scratch wound healing tests. The scratch images were taken from 0 to 24 hours after scratching showing that the migration of A375 (Wt and RIPK4.KO) cells into the cell-free area (indicated by the yellow line) is significantly slowed in the presence of 1,25-D3 (100 nM) compared to EtOH (treatment vehicle) controls (top panel). Bar graph illustrating the percentage wound closure at the indicated time points during the wound scratch test, n = 3 (bottom panel).

**
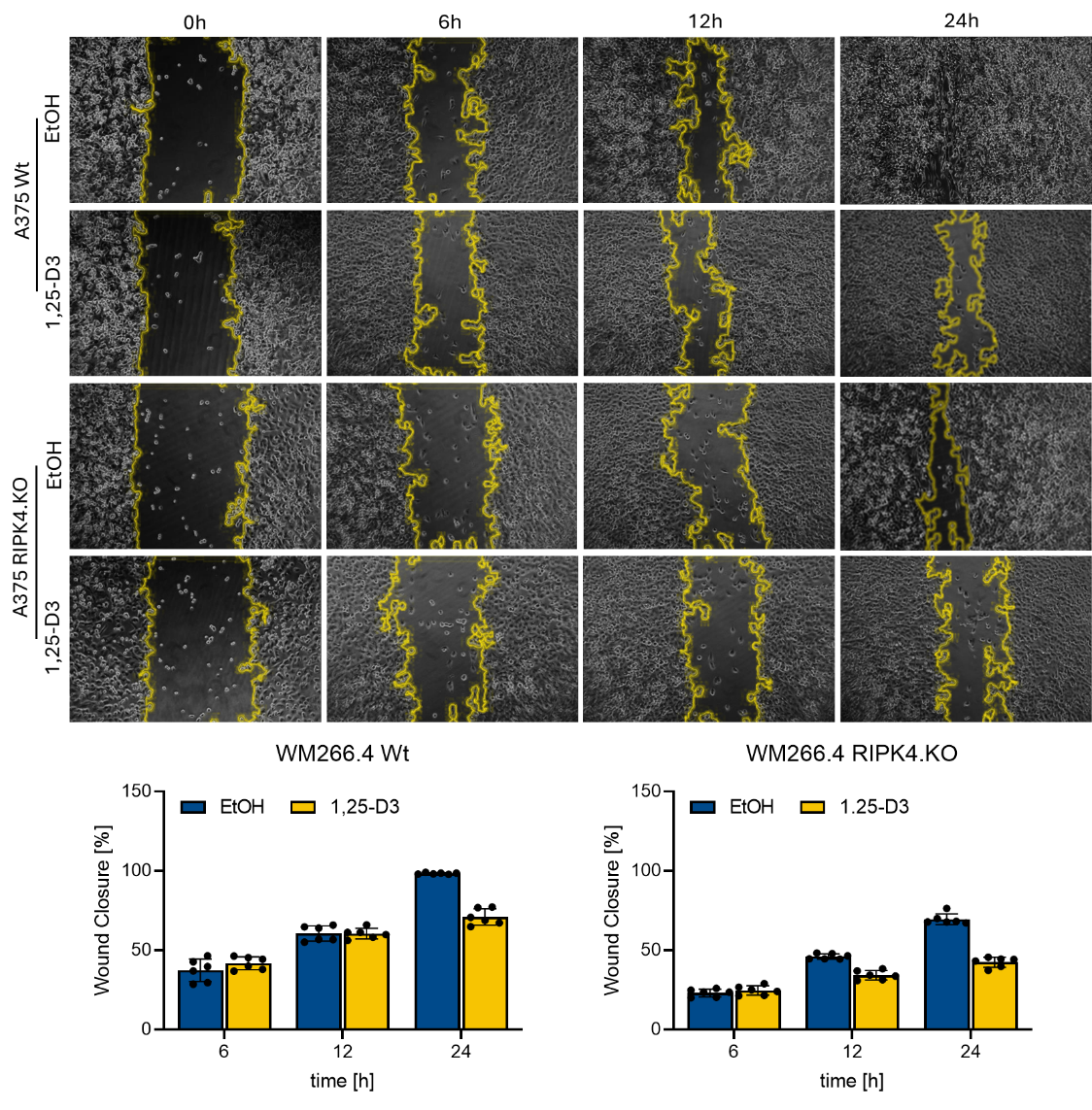
**

**Figure S4.** Images from *in vitro* scratch wound healing tests. The scratch images were taken from 0 to 24 hours after scratching showing that the migration of WM266.4 (Wt and RIPK4.KO) cells into the cell-free area (indicated by the yellow line) is significantly slowed in the presence of 1,25-D3 (100 nM) compared to EtOH (treatment vehicle) controls (top panel). Bar graph illustrating the percentage wound closure at the indicated time points during the wound scratch test, n = 3 (bottom panel).

**
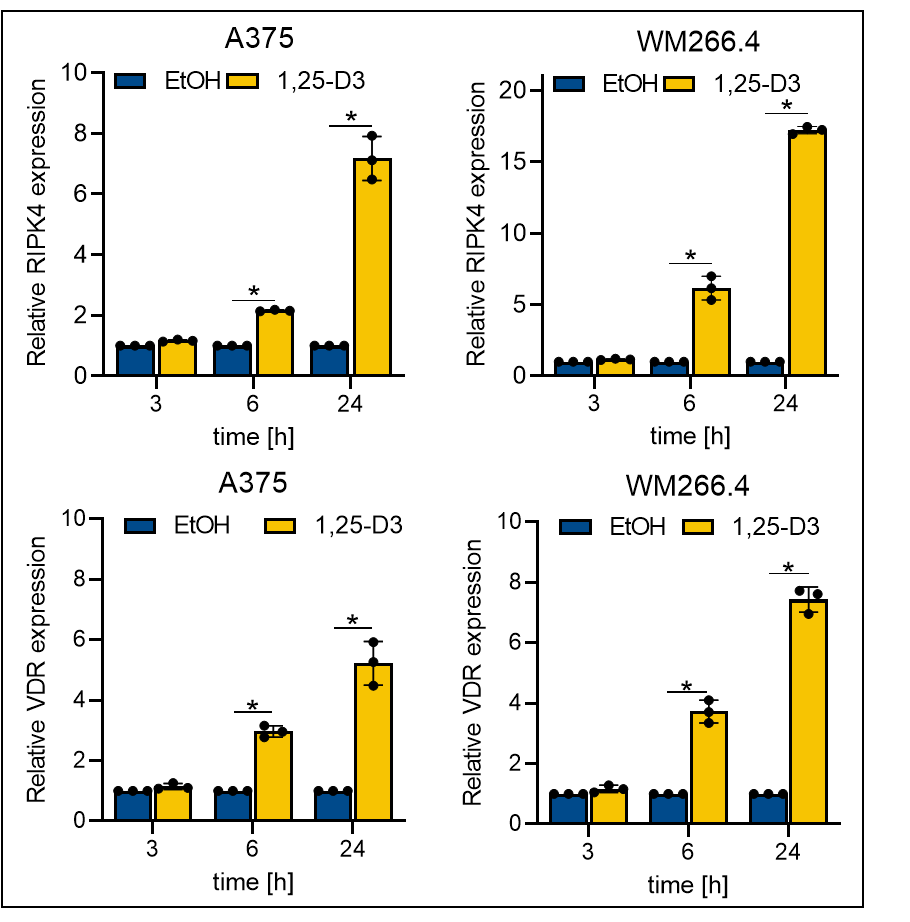
**

**Figure S5.** The effect of 1,23-D3 on the expression of RIPK4 and VDR. Transcript levels of RIPK4 in A375 and WM266.4 cells normalized to GAPDH at indicated time point after the treatment with 1,25-D3 (100 nM) or EtOH as a vehicle control; n = 3. * p < 0.05 were considered significant.


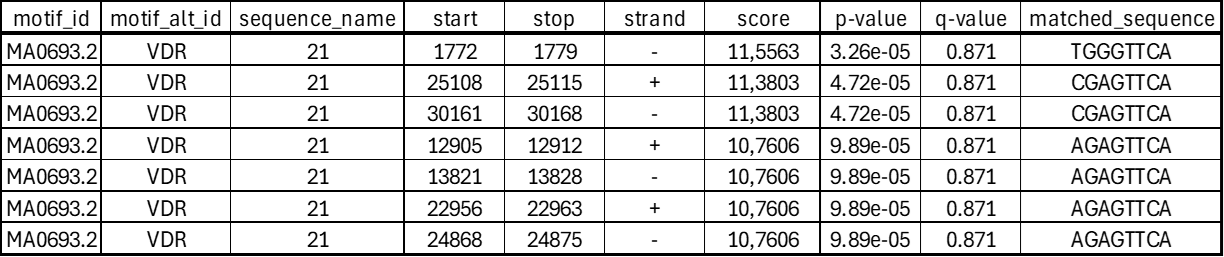


**Table S1.** Bioinformatics analysis of the RIPK4 promoter region. Probability of occurrence of VDRE-elements in the 2000 bp sequence upstream of RIPK4 promoter gene were tested using Find Individual Motif Occurrences (FIMO) tool (<http://meme.sdsc.edu>). The sequences for VDRE were downloaded from <https://jaspar.elixir.no>.

**
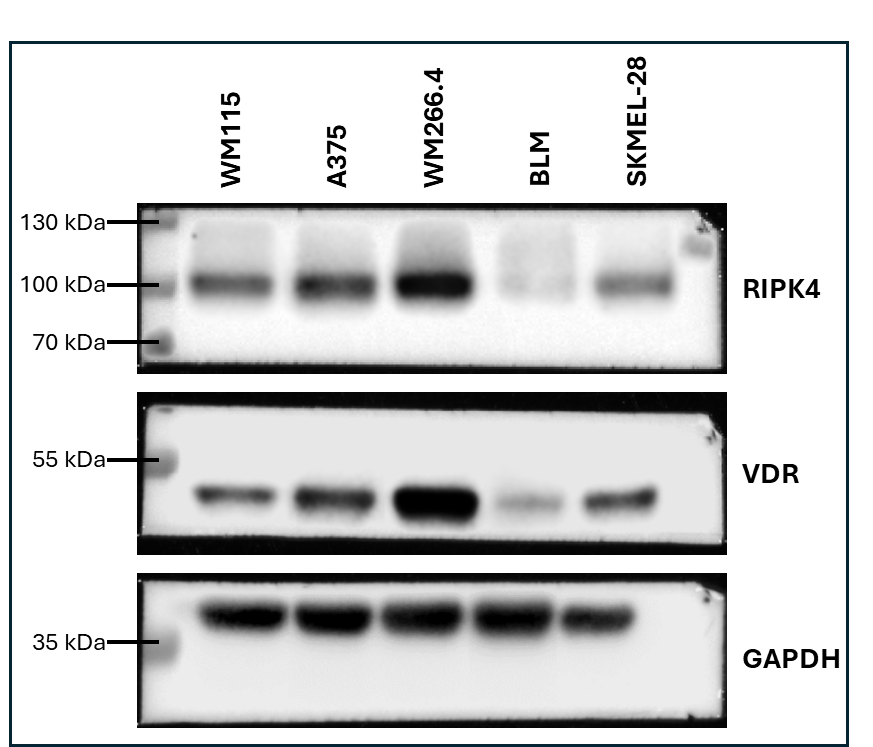
**

**Figure S6.** Original unedited blot related to Figure 1. Membrane was cut before hybridization with antibodies.


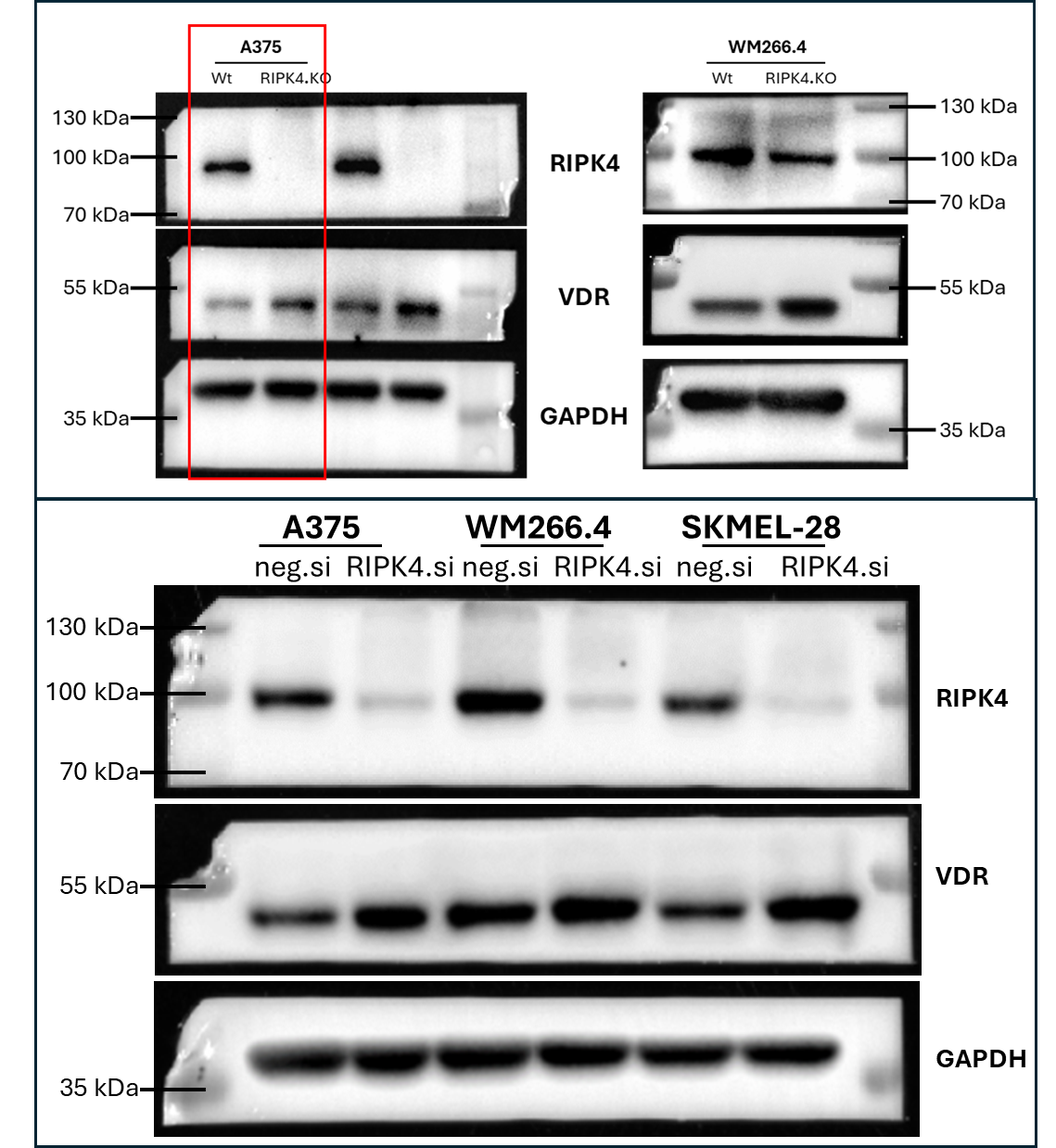


**Figure S7.** Original unedited blot related to Figure 2 A (upper panel), and B (lower panel). Membrane was cut before hybridization with antibodies.


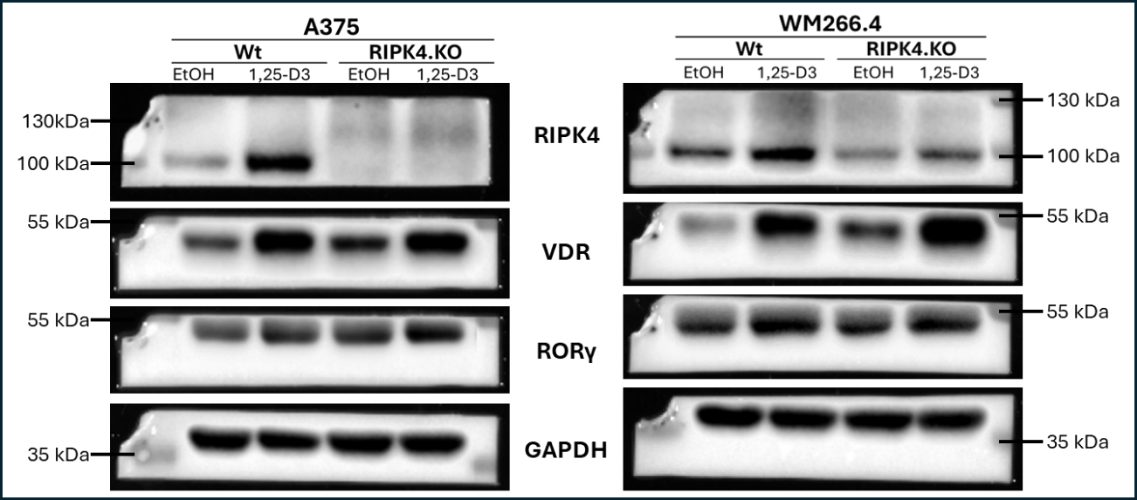


**Figure S8.** Original unedited blot related to Figure 5. Membrane was cut before hybridization with antibodies.


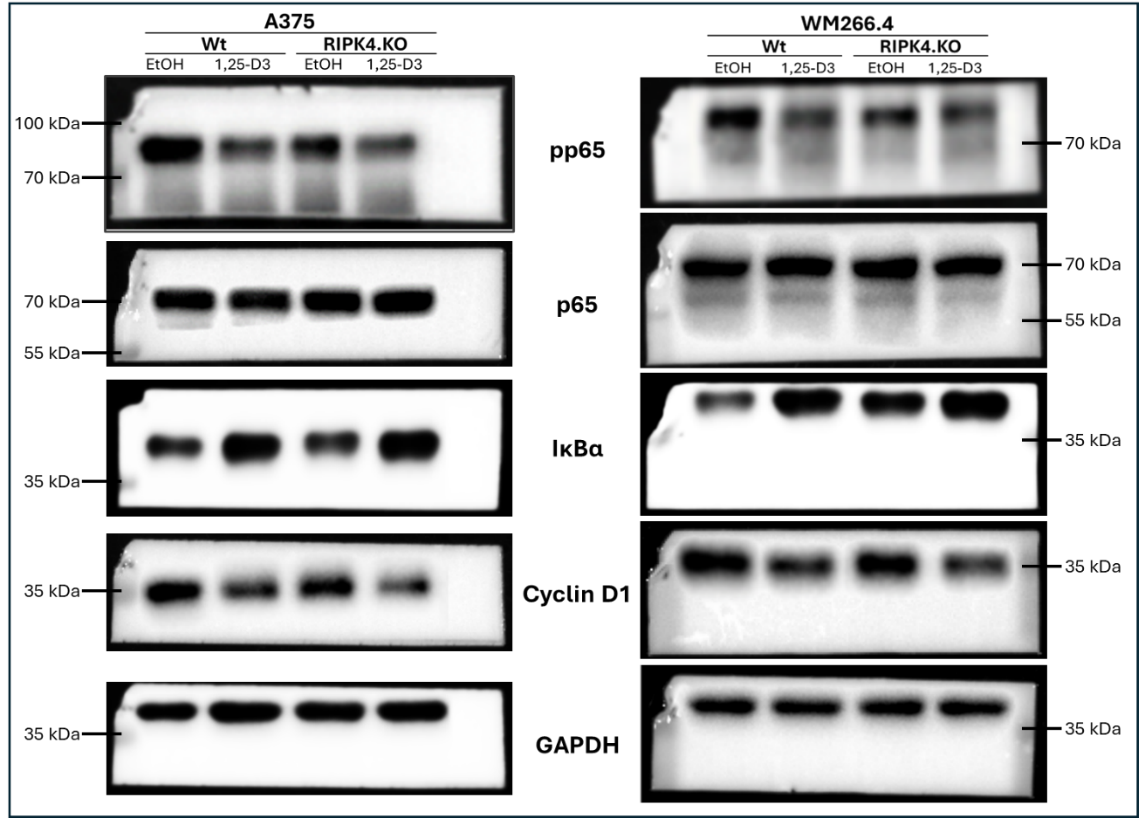


**Figure S9.** Original unedited blot related to Figure 7. Membrane was cut before hybridization with antibodies.
